# The Effect of Permethrin Resistance on *Aedes aegypti* Transcriptome Following Ingestion of Zika Virus Infected Blood

**DOI:** 10.3390/v10090470

**Published:** 2018-09-01

**Authors:** Liming Zhao, Barry W. Alto, Dongyoung Shin, Fahong Yu

**Affiliations:** 1 Florida Medical Entomology Laboratory, University of Florida, 200 9th Street South East, Vero Beach, FL 32962, USA; bwalto@ufl.edu (B.W.A.); dshin@ufl.edu (D.S.); 2 Interdisciplinary Center for Biotechnology Research, University of Florida, 2033 Mowry Road, Gainesville, FL 32611, USA; fyu@ufl.edu

**Keywords:** *Aedes aegypti*, RNA-seq, insecticide resistance, Zika virus, detoxification and immune system responses

## Abstract

*Aedes aegypti* (L.) is the primary vector of many emerging arboviruses. Insecticide resistance among mosquito populations is a consequence of the application of insecticides for mosquito control. We used RNA-sequencing to compare transcriptomes between permethrin resistant and susceptible strains of Florida *Ae. aegypti* in response to Zika virus infection. A total of 2459 transcripts were expressed at significantly different levels between resistant and susceptible *Ae. aegypti*. Gene ontology analysis placed these genes into seven categories of biological processes. The 863 transcripts were expressed at significantly different levels between the two mosquito strains (up/down regulated) more than 2-fold. Quantitative real-time PCR analysis was used to validate the Zika-infection response. Our results suggested a highly overexpressed P450, with AAEL014617 and AAEL006798 as potential candidates for the molecular mechanism of permethrin resistance in *Ae. aegypti*. Our findings indicated that most detoxification enzymes and immune system enzymes altered their gene expression between the two strains of *Ae. aegypti* in response to Zika virus infection. Understanding the interactions of arboviruses with resistant mosquito vectors at the molecular level allows for the possible development of new approaches in mitigating arbovirus transmission. This information sheds light on Zika-induced changes in insecticide resistant *Ae. aegypti* with implications for mosquito control strategies.

## 1. Introduction

*Aedes aegypti* (L.) is the primary vector of emergent mosquito-borne viruses, including yellow fever, dengue, chikungunya, and Zika [1,2]. Zika fever is an emerging viral disease (family Flaviviridae, genus *Flavivirus*) that is transmitted to humans by infected female mosquitoes, primarily *Ae. aegypti* and *Ae. albopictus*. Zika virus (ZIKV) consists of three lineages, one from Asia and two from Africa [3]. Molecular analyses indicate that ZIKV originated in Uganda and spread to Central and West Africa through two introductions occurring in 1935 and 1940 [3]. Zika spread eastward to Asia around 1945 [3]. The Asian lineage of ZIKV was responsible for the first epidemic on Yap Island, Micronesia in 2007 followed by another outbreak in French Polynesia during 2013 [4]. Zika was detected in northeastern Brazil in early 2015 resulting in 1.5 million human cases [5,6]. Since the arrival of Zika in Brazil, the mosquito-borne pathogen has spread throughout the Americas and local transmission in the U.S. is a major public health risk among parts of the Gulf Coast. Symptoms associated with Zika infection are only observed in 20% of cases, and symptoms are often mild including fever, rash, joint pain, conjunctivitis, headache, and muscle pain. However, ZIKV is strongly associated with more severe outcomes including birth defects, such as microcephaly [7], and neurological complications, such as Guillain–Barré syndrome [8].

Recurrent use of insecticides in mosquito control and agricultural pest control has selected for insecticide resistance in mosquito populations [9,10,11,12,13,14,15]. Permethrin resistance is widespread in *Ae. aegypti* which compromises mosquito control and disease prevention efforts [11,13]. There are several modes that mosquito populations have become resistant to insecticides in nature. Insecticide resistance mechanisms can be divided into penetration resistance, behavioral resistance, target-site insensitivity, and metabolic detoxification of insecticides [16,17]. Penetration resistance occurs when insects absorb an insecticide more slowly than susceptible insects attributable to cuticle barriers. Behavioral resistance occurs when insects recognize and alter their behavior (feeding and movement) in the presence of an insecticide. Target-site insensitivity results from modifications (e.g., point mutations in genes encoding target proteins) to sites where the insecticide binds to reduce the detrimental effects of an insecticide. Metabolic detoxification of insecticides results when resistant insects detoxify or destroy insecticides more effectively than susceptible insects. Metabolic detoxification of insecticides is one of the most common mechanisms of resistance. Detoxification of insecticides in mosquitoes include three major gene families: Cytochrome P450s, esterases, and glutathion S-transferases (GSTs) [18,19].

Pyrethroid/permethrin resistant mosquitoes exhibit insecticide resistance through elevated levels of multiple detoxification enzymes, including GSTs [14,20,21,22], ATP-binding cassette (ABC) transporters [21,23], carboxylesterase [14,24,25], and cytochrome P450 [14,26,27,28,29,30,31,32,33]. Penetration resistance occurs through modifications in cuticular proteins [14,34]; metabolic resistance occurs by detoxification enzymes and G-protein-coupled receptor [26], UDP glucuronosyltransferase, and glucosyl/glucuronosyl transferase [32,35]; and target site insensitivity is mediated by changes in the voltage-gated sodium channel gene [11,15,36,37], and transcription factor Maf-S [38].

Multigene expression in response to arbovirus infection has been reported in *Ae. aegypti* and other species of mosquitoes [39,40,41,42,43]. Many genes are involved in the mosquito’s antiviral immunity, such as antimicrobial peptide (AMP)-coding genes [44]. Immune responses and several arthropod immunity pathways such as Toll, Imd, JAK/STAT, and RNAi also play important roles during mosquito arboviral infection [39,41,42,45]. In addition to antiviral responses, several studies have reported changes in expression levels of multiple categories of biological processes in response to ingestion of arbovirus infected blood. An infection study showed that trypsins, metalloproteinases, and serine-type endopeptidases were significantly upregulated in mosquitoes following ingestion of chikungunya virus infected blood [39]. Along the same lines, another study reported that dengue virus infection induced upregulation of gene expression associated with lipogenesis, lipolysis and fatty acid β-oxidation, and lipid metabolism [46].

The unprecedented global spread of ZIKV has created a need to improve our understanding of host–microbe interactions in this mosquito–arbovirus system [43,45,47,48]. Understanding the mechanism(s) of insecticide resistance may provide insight into novel molecular strategies that may be used to improve control of Zika vector. To understand mechanisms by which pyrethroid/permethrin resistant *Ae. aegypti* populations alter their gene expression in response to ZIKV infection, we used RNA-sequencing (RNA-seq) and functional analysis to explore the difference between a permethrin resistant *Ae. aegypti* population (Key West, FL, USA) and a permethrin susceptible population (Orlando, FL, USA). Zika infection activated metabolic pathways (e.g., drug metabolism) in which some transcripts were putatively linked to insecticide resistance. Our observations provide a global picture of gene expression associated with metabolic detoxification among permethrin resistant and susceptible populations of *Ae. aegypti*, including antiviral responses following ingestion of ZIKV. This study aims to improve our understanding of the entomological components of ZIKV epidemiology in context of insecticide-based control through a combination of traditional genetic and biochemical approaches to address issues related to mosquito vector control.

## 2. Materials and Methods

### 2.1. Mosquito Strains

*Ae. aegypti* larvae were collected from Key West (24.55° N, 81.78° W), Florida, USA and maintained at the Florida Medical Entomological Laboratory (FMEL) in Vero Beach, FL since 2011. The parental collection of *Ae. aegypti* from the field was initially tested for permethrin resistance, then subjected to permethrin selection for 15 generations (see below) and again assayed for resistance (referred to as the resistant strain). Assays for resistance followed WHO protocols for mortality thresholds using the permethrin CDC bottle bioassay with a diagnostic dose and mortality rate (>90%) in laboratory bioassays using modified WHO bottle bioassay (WHO 2016). Bottles used in the assays were coated with a known amount of permethrin (diagnostic dose, 47 µg/bottle), after which adult *Ae. aegypti* mosquitoes were placed in the bottle and observed for 2 h and mortality was recorded.

The Orlando strain of *Ae. aegypti* was collected from Orlando (28.53° N, 81.37° W), Florida, USA and reared in the Mosquito and Fly Research Unit, Center for Medical, Agricultural and Veterinary Entomology, ARS-USDA in Gainesville, FL since 1952. The Orlando strain is recognized as a permethrin susceptible strain of *Ae. aegypti* [9].

### 2.2. Zika Virus Infection 

Four-day-old female adults were fed defibrinated bovine blood containing either ZIKV (treatment) or blood lacking virus (control). The method utilized in this study was as previously described by Zhao et al. [48]. Mosquitoes were deprived of sucrose, but not water, 24 h before blood feeding trials performed in a biosafety level-3 laboratory at the FMEL. Isolates of the Asian lineage of ZIKV (strain PRVABC59, GenBank accession # KU501215.1) from Puerto Rico were prepared in African green monkey (Vero) cells and used in the mosquito infection study. Monolayers of Vero cells were inoculated with 500 µL of diluted stock virus (multiplicity of infection, 0.1) and incubated for 1 h at 37 °C and 5% CO_2_ atmosphere, after which 24 mL media (M199 medium supplemented with 10% fetal bovine serum, penicillin/streptomycin, and mycostatin) were added to each tissue culture flask and incubated for six days for propagation of ZIKV. Freshly harvested media from infected cell cultures were combined with defibrinated bovine blood and ATP (0.005 M) and presented to mosquitoes using a membrane feeding system (Hemotek, Lancashire, UK) for one hour feeding trials. Control blood meals were prepared similarly except that monolayers of Vero cells were inoculated with media only. Samples of infected blood were collected at the time of the feedings and stored at −80 °C for later determination of virus titer. Mosquitoes were fed 6.4 log10 plaque forming units (pfu)/mL of ZIKV (Table 1). The experiments were replicated three times. The ZIKV infected and control mosquitoes from two *Ae. aegypti* strains were harvested for a time course study. Individual mosquitoes were dissected into body and legs and tested to confirm susceptibility to infection and disseminated infection rates, respectively. Ten mosquitoes (12 h and 7 days post ZIKV infection) were pooled for each sample for RNA sequencing.

### 2.3. RNA Extraction

All samples (10 mosquitoes per pool) were homogenized with a plastic pestle in the 1 mL TRIzol reagent (Ambion, Life Technologies, Carlsbad, CA, USA). Total RNAs were isolated using TRIzol reagent according to the manufacturer’s instruction and followed a standard protocol (Ambion, Life Technologies). To avoid genomic DNA contamination, the RNA samples were processed by DNase I (RNase-free) following the manufacturer’s instructions (Thermo Scientific, Wilmington, DE, USA). The RNA samples were quantitated by NANODROP 2000 Spectrophotometer (Thermo Scientific, Wilmington, DE, USA).

### 2.4. RNA-Seq Library Preparation and Sequencing

Preparation and sequencing libraries were carried out in the Interdisciplinary Center for Biotechnology Research (ICBR), at the University of Florida following the manufacturer’s protocol (Illumina, Inc., San Diego, CA, USA). The TruSeq DNA Library Preparation Kit (Illumina, Inc., San Diego, CA, USA) was used to prepare DNA libraries with insert sizes from 300–500 bp for high-throughput sequencing. For the Illumina NextSeq 500 run, the NCS v1.2 control software was used. The libraries were pooled at equimolar concentrations to yield a 4 nM stock solution, containing 0.33 nM of each library. The library pool was prepared for sequencing following the manufacturer protocol. The Illumina^®^ NextSeq^®^ 500 sequencing platform was used to create paired-end reads using Illumina’s sequencing-by-synthesis approach (Illumina^®^, San Diego, CA, USA) using 2 × 150 cycles.

### 2.5. Data Mining and RNA-Seq Analysis

Reads acquired from the sequencing platform were cleaned up with the Cutadapt program (Martin 2011) to trim off sequencing adaptors, low quality bases, and potential errors introduced during sequencing or library preparation. Reads with a quality phred-like score <20 and read length <40 bases were excluded from RNA-seq analysis.

The transcripts of *Ae. aegypti* (18,840 sequences) were retrieved from the VectorBase (https://www.vectorbase.org/organisms/aedes-aegypti/liverpool) and used as reference sequences for RNA-seq analysis. The cleaned reads of each sample were mapped independently to the reference sequences using the bowtie2 mapper (version. 2.2.3) with a “3 mismatches a read” allowance [50]. The mapping results were processed with the samtools and scripts developed in house at ICBR to remove potential PCR duplicates and to choose uniquely mapped reads for gene expression analysis. Gene expression was assessed by counting the number of mapped reads for each transcript [51]. Significant up- and downregulated genes were selected using the adjusted *p*-value (p-adj), log2 fold-change (log2FC), or both for the analysis. The RNA-seq data have been deposited to NCBI (Accession number: GSE118858, https://www.ncbi.nlm.nih.gov/gds/?term=GSE118858).

### 2.6. Assignments of Gene Ontology (GO) Terms and Pathway Analyses

All genes with p-adj ≤ 0.01 were selected for the GO enrichment analysis (http://amigo.geneontology.org/amigo). The GO terms of *Ae. aegypti* genes were retrieved from the VectorBase and assigned to GO hierarchies and functional groups. The genes matched to the functional categories of immune system process (GO:0002376), response to stimulus (GO:0050896), developmental process (GO:0032502), cellular process (GO:0009987), signal transducer activity (GO:0004871), biological regulation (GO:0065007), electron carrier activity (GO:0009055), transporter activity (GO:0005215), catalytic activity (GO:0003824), and metabolic process (GO:0008152) were divided into two pools: the downregulated and upregulated gene pools based on the log2 transformed-fold-change of the RNA-seq results. The selected genes that were not assigned GO terms or categorized to other functional groups were treated as the unknown group.

### 2.7. C-DNA Synthesized and qPCR Amplification 

C-DNA synthesis was performed using methods described by Zhao 2017 et al. [48]. The qPCR assay for confirming genes in *Ae. aegypti* was performed using Platinum^®^ SYBR^®^ Green qPCR SuperMix-UDG with ROX (Invitrogen, Carlsbad, CA, USA) in a volume of 15 µL on a BIO-RAD C1000 Touch Thermal Cycler, CFX 96™ Real-Time System (“Bio-Rad”). The primers were designed using Primer3 program https://sourceforge.net/projects/primer3 (Table S1).

## 3. Results

### 3.1. Global Changes in Transcriptome of the Aedes Aegypti Female Adult in Response to ZIKV Infection

**Figure 1:**
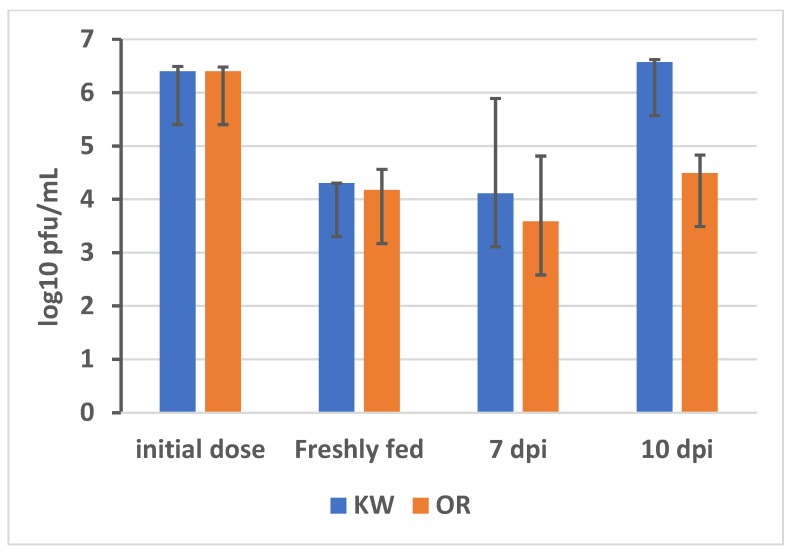
Zika virus titers in infectious blood meals and blood fed mosquitoes for permethrin resistant (KW) and susceptible (OR) strains of *Aedes aegypti*, including initial dose in bloodmeal, freshly fed, 7 days post infection (7 dpi), and 10 days post infection (10 dpi). Zika virus (strain PRVABC59, GenBank accession # KU501215.1) isolated from a human infected in Puerto Rico in 2015.

To understand the molecular interactions of the arbovirus with permethrin resistant *Ae. aegypti* from Florida, RNA-seq was conducted to explore the global changes in the *Ae. aegypti* (Key West and Orlando strains) transcriptome in response to oral ingestion of ZIKV infected blood and ZIKV infection. In this study, four-day-old female *Ae. aegypti* adults were fed a blood meal containing 6.4 log10 pfu/mL of ZIKV (Figure 1). Fresh fed mosquitoes ingested 4.2 to 4.3 log10 pfu/mL of ZIKV. By 7 days post infection (dpi), ZIKV titer in mosquito bodies were 4.1 ± 1.7 log10 pfu/mL and 3.6 ± 1.2 log10 pfu/mL for the permethrin resistant and susceptible strains of *Ae. aegypti*, respectively. A two-tailed *t*-test showed no significant differences in ZIKV titer in the bodies of the two strains of *Ae. aegypti* (t17 = 0.77, *p* = 0.44). By 10 dpi, ZIKV titer in permethrin resistant strain mosquito bodies were 6.5 ± 0.05 log10 pfu/mL, which was 100-fold higher (t4 = 8.12, *p* = 0.001) than the titer of the susceptible strain (4.5 ± 0.34 log10 pfu/mL). This result demonstrated that the ZIKV replication rates were higher at this point in the infection process for the permethrin resistant strain than the susceptible strain.

**Figure 2:**
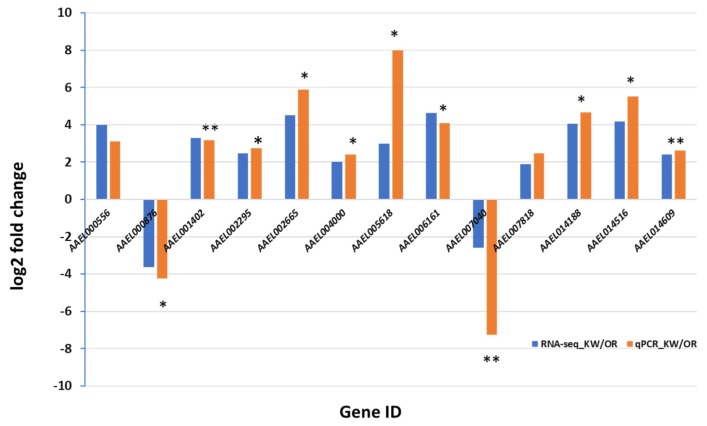
Validation of the expression of transcripts between the permethrin resistant (KW) and susceptible (OR) strains of *Aedes aegypti* by qRT-PCR. * *p* < 0.05. ** *p* < 0.01.

Twelve hours post infection and 7 dpi, RNAs from female *Ae. aegypti* were extracted. A total of 24 RNA-seq libraries were created from *Ae. aegypti* infected by ZIKV (12 h and 7 dpi) and control (fed uninfected blood, 12 h and 7 dpi). Three replicates of each group were prepared and sequenced. A total of 706,051,842 raw reads were generated from the permethrin resistant and susceptible strains. The cleanup resulted in 705,983,440 cleaned reads, which mapped to 18,840 transcripts of *Ae. aegypti* (Table S2). The qPCR of the selected 13 genes showed significantly different expression levels between the two *Ae. aegypti* strains in response to ZIKV at 7 dpi, supporting the RNA seq data analysis (Figure 2).

#### 3.1.1. Expression Profiles of Differentially Expressed (DE) Transcripts in Response to Blood Feeding (Control) between Two *Aedes aegypti* Strains, Resistant Versus Susceptible Strains

**Figure 3:**
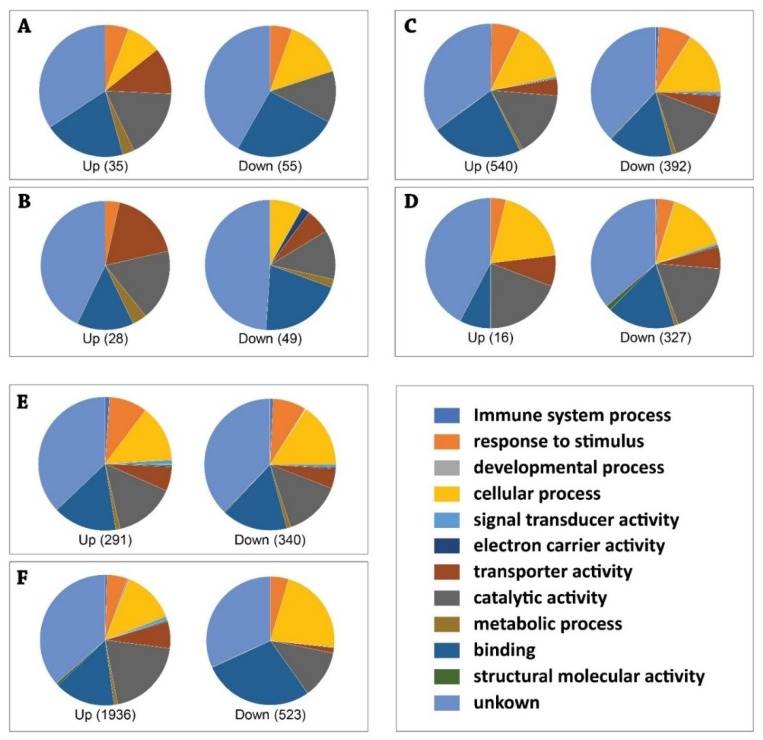
Overview of the functional categories of differentially expressed (DE) transcripts in response to ZIKV infection and between the permethrin resistant (KW) and susceptible (OR) strains of *Aedes aegypti* blood-feeding control. DE transcripts were determined based on statistical analysis by DESeq package. The total number of DE transcripts for each comparison is shown in parentheses in each figure. Gene ontology analysis of DE genes was performed based on the database of AmiGO 2 (http://amigo.geneontology.org/amigo), and pie charts were generated using Excel. Up, upregulated DE genes; Down, downregulated DE genes. Please also notice the details in the Supplementary Figure S1. GO analyses for RNA-seq data. (**A**) 12 h post injection KW-Control compared with OR-Control; (**B**) 12 h post infection, KW-ZIKV compared with OR-ZIKV; (**C**) 7 dpi, KW-ZIKV compared with KW-Control; (**D**) 7 dpi, OR-ZIKV compared with OR-Control; (**E**) 7 dpi, KW-Control compared with OR-Control; (**F**) 7 dpi, KW-ZIKV compared with OR-ZIKV.

Functional analysis based on Gene Ontology were conducted on the significant differentially expressed (DE) transcripts between the permethrin resistant and susceptible strains of *Ae. aegypti*. Comparison of the transcriptome profiles showed a relatively low number of DE transcripts 12 h after blood-feeding. There were 90 DE transcripts at 12 h post blood-feeding (p-adj ≤ 0.01), of which 35 were upregulated and 55 were downregulated (Figure 3A and Figure S1A). The largest proportion of total number of DE genes (38.9%) had unknown functions (Figure 3A and Figure S1A). Other DE transcripts mainly belonged to the functional categories of Binding (23.3%), Catalytic activity (14.4%), Cellular process (12.2%), Response to stimulus (5.6%), and Transporter activity (4.4%). All other categories were less than 1%. After 7-days post blood-feeding, 631 DE genes were significantly different, (p-adj ≤ 0.01; 291 upregulated and 340 downregulated) (Figure 3E and Figure S1E). Of those 631 transcripts, 36.8% of those genes were assigned to an unknown function (Figure 3E and Figure S1E). Other DE genes were mainly placed in functional categories of Binding (20.0%), Catalytic activity (13.9%), Cellular process (14.1%), Response to stimulus (7.9%), and Transporter activity (4.8%).

#### 3.1.2. Expression Profiles of DE Transcripts in *Aedes Aegypti*, Resistant and Susceptible Strains, in Response to ZIKV

Analysis of mRNA expression profiles of *Ae. aegypti* mosquitoes at different time points of ZIKV infection revealed a relatively low number of DE transcripts 12-h after blood-feeding. Only five DE transcripts were identified in the susceptible strain and none in the resistant strain. However, there were 932 DE genes (p-adj ≤ 0.01; 540 upregulated and 392 downregulated) in the resistant strain of *Ae. aegypti* at 7 dpi with ZIKV (Figure 3C and Figure S1C). Most of these transcripts (36.2% in the total: 35.0% in the Up; 37.8% in the Down) had unknown functions. The remaining of the DE transcripts matched to the functional categories of Binding (19.5% in the total: 22.0% in the Up; 16.1% in the Down), Catalytic activity (15.0% in the total: 15.7% in the Up; 14.1% in the Down), Cellular process (14.95 in the total: 14.3% in the Up; 15.8% in the Down), Response to stimulus (7.5% in the total: 7.0% in the Up; 8.2% in the Down), and Transporter activity (4.4% in the total: 4.1% in the Up; 4.8% in the Down). All other categories were lower than 1%. About 57.9% of 932 DE transcripts were upregulated in the *Ae. aegypti* resistant strain in response to the ZIKV infection at 7 dpi.

Functional analysis based on the significant DE transcripts between the ZIKV exposed susceptible strain and control susceptible at 7 dpi showed that most of the transcripts were downregulated (p-adj ≤ 0.01; 26 upregulated and 327 downregulated) (Figure 3D and Figure S1D). Approximately 36.8% of the DE transcripts (42.3% in the Up; 36.4% in the Down) had unknown functions. The other DE transcripts were categorized into the functional groups of Binding (16.4% in the total: 7.7% in the Up; 16.5% in the Down), Catalytic activity (18.1% in the total: 19.2% in the Up; 18.0% in the Down), Cellular process (15.3% in the total: 19.2% in the Up; 15.0% in the Down), Response to stimulus (4.2% in the total: 3.8% in the Up; 4.3% in the Down), and Transporter activity (5.6% in the total: 7.7% in the Up; 5.5% in the Down). All other categories were lower than 1%. Most of the DE transcripts (92.6% of 353 transcripts) were downregulated in the susceptible strain in response to the ZIKV infection at 7 dpi.

#### 3.1.3. Expression Profiles of DE Transcripts in Response to ZIKV Infection between Two Strains *Aedes Aegypti*, Resistant Versus Susceptible Strains

Analysis and comparison of mRNA expression profiles of *Ae. aegypti* at different strains following ZIKV infection revealed that ZIKV induced a relatively low number of DE transcripts 12 h after blood-feeding. We observed 77 DE transcripts (p-adj ≤ 0.01), of which 28 were upregulated and 49 were downregulated (Figure 3B and Figure S1B) at 12 h post infection. Among those DE transcripts, 46.8% in total (42.9% in the UP; 49% in the Down) had unknown functions. The other DE transcripts mainly belonged to the functional categories of Binding (18.2% in the total: 14.3% in the Up; 20.4% in the Down), Catalytic activity (14.2% in the total: 17.9% in the Up; 12.2% in the Down), Cellular process (5.2% in the total: 0% in the Up; 8.2% in the Down), and Transporter activity (10.4% in the total: 17.9% in the Up; 6.1% in the Down). All other categories were lower than 1%. Most of the DE genes (63.6%) were downregulated in the *Ae. aegypti* resistant strain compared to the susceptible strain at the 12 h post infection.

Comparison of the transcriptome profiles of two *Ae. aegypti* strains in response to ZIKV 7 dpi revealed 2459 DE transcripts (p-adj ≤ 0.01; 1936 upregulated and 523 downregulated, Figure 3F and Figure S1F). Most of those DE transcripts (35.5% in the total) had unknown functions (Figure 3F and Figure S1F). Of the DE transcripts that were up regulated in the resistant strain, 36.6% had unknown functions; while 31.7% of the downregulated DE transcripts were of unknown function. The remaining DE transcripts matched the functional categories of Catalytic activity (18.2% in the total: 19.8% in the Up; 12.0% in the Down), Cellular process (15.0% in the total: 13.7% in the Up; 22.0% in the Down), Response to stimulus (5.0% in the total: 5.1% in the Up; 4.5% in the Down), and Transporter activity (5.5% in the total: 6.6% in the Up; 1.3% in the Down). All other categories were lower than 1%. Most of the DE transcripts (78.7%) were upregulated in the *Ae. aegypti* resistant strain compared to the susceptible strain at the 7 dpi. The data showed global changes in the two strains of *Ae. aegypti* female adult transcriptome in response to ZIKV infection.

#### 3.1.4. DE Transcripts Related to Immunity in Response to ZIKV Infection

When *Ae. aegypti* were infected with ZIKV at 7 dpi, a total of 863 transcripts had 2-fold or more changes (p-adj ≤ 0.01; log2 fold change > ±2.0). Seventy-one immunity-related DE transcripts were significantly upregulated in response to ZIKV 7 dpi between the two strains. These results suggest that ingestion of ZIKV can induce an immune response in the permethrin resistant Key West strain (Table S3A). These upregulated immunity related genes encoded two allergens, one caspase-1, eleven Clip-domain serine protease family B and D, four C-type lectins, two C-type lysozymes, one cysteine-rich protein, one cysteine-rich venom protein (AAEL005098, 5.77 log2 fold change), one environmental stress-induced protein, five fibrinogen and fibronectins, two Gram-negative binding proteins (GNBP), one granzyme A precursor, one lachesin, thirteen leucine-rich immune proteins, one M protein, one neuroendocrine protein, one p37NB protein, one peptidoglycan recognition protein (AAEL012380), one prophenoloxidase (AAEL011763), one rh antigen, SEC14, SEC15, SEC16, one thioester-containing protein (tep2), one toll protein and four Toll-like receptors, eight trypsins, and three venom allergens (Table S3A).

Compared with the control group, more immune related enzymes at 7 dpi infected with ZIKV were detected and most of them were upregulated significantly (Table S3A,B). The comparison between *Ae. aegypti* infected with ZIKV and the control at the 7-dpi in the resistant strain revealed that 318 transcripts had changes of 2-fold or more in either direction. Fifteen DE transcripts related to immunity were significantly dysregulated more than 2-fold (seven upregulated and eight downregulated, Table S3C). These transcripts encoded two Class C Scavenger Receptors, two Clip-domain serine proteases family B, two C-type lectins, one cysteine-rich venom protein (AAEL005098, 2.71 log2 fold change), one Gram-negative binding protein, one lachesin, three leucine-rich transmembrane proteins, a shoc2, one venom allergen, and one Wnt10a protein (Table S3C). In the Orlando strains infected with ZIKV at 7 dpi, a total 128 transcripts had changes of 2-fold or more, but only one was upregulated. All 14 DE transcripts related to immunity between ZIKV infected and the control group at 7 dpi were significantly downregulated (Table S3D). These transcripts encoded six Clip-domain serine proteases family B, one C-type lectin, five leucine-rich immune proteins, one Trypsin 3A1 precursor, and one tyrosine kinase receptor (Table S3D). Both the *Ae. aegypti* resistant and susceptible strains infected with ZIKV at the 7 dpi shown regulated with Clip-domain serine protease family B, C-type lectin, and some leucine-rich proteins.

Some important immunity transcripts were significantly upregulated (more than 4-fold) in the permethrin resistant than the susceptible strains, such as prophenoloxidase and M protein. The prophenoloxidase (AAEL011763) is a modified form of the complement response found in insects, and a major innate defense system in invertebrates that controls the melanization of pathogens and damaged tissues [52]. M protein (AAEL011747), a strongly antiphagocytic and a major virulence factor in viruses, parasites, and bacteria aids in entering by counteracting the mosquito’s defenses [53,54]. In addition, two lachesins, a novel immunoglobulin superfamily protein required for morphogenesis of the Drosophila tracheal system, were also significantly upregulated [55]. The peptidoglycan recognition protein (AAEL012380), an important role in the innate immune response, was correspondingly upregulated significantly in the Key West strain [56].

#### 3.1.5. DE Transcripts Related to Detoxification in Response to ZIKV Infection

The RNAseq study between two *Ae. aegypti* strains infected with ZIKV at 7 dpi showed that 62 DE transcripts related to detoxifications were upregulated more than 2-fold in response to ZIKV. These transcripts encoded one alcohol dehydrogenase, two aldehyde oxidases, one aldo-keto reductase, two Carboxy/choline esterases, one core 1 UDP-galactose galactosyltransferase, 33 cytochrome P450, one d-amino acid oxidase, one epoxide hydrolase, seven glucosyl/glucuronosyl transferases, one glutamate semialdehyde dehydrogenase, three n-acetylgalactosaminyltransferases, one prophenoloxidase, four short-chain dehydrogenases, one sterol desaturase, and one thioredoxin peroxidase (Table S4A).

Four cytochrome P450 (AAEL009018, AAEL014609, AAEL014617, and AAEL014893) were reported as associated with insecticide resistance in several populations of *Ae. aegypti* [31,32,57]. Compared with the control, between the Key West strain and the Orlando strain at 7 dpi infected with ZIKV, more detoxification enzymes were detected and most of them were upregulated significantly more than 2-fold (Table S4A,B), suggesting those genes might associate with insecticide resistance.

Comparing the Key West *Ae. aegypti* infected with ZIKV with the Key West control at the 7 dpi, 19 DE transcripts related to detoxification were significantly regulated (11 upregulated and eight downregulated, Table S4C). Nevertheless, all 14 DE transcripts related to detoxification were significantly downregulated between the Orlando *Ae. aegypti* infected with ZIKV and the Orlando control at the 7 dpi (Table S4D).

According to previous studies [31,32,57], 23 transcriptions of detoxification enzymes associated with permethrin resistance were significantly upregulated at 7 dpi between the Key West strain and the Orlando strain in response to ZIKV infection (Table 1A). They encoded an alcohol dehydrogenase (AAEL012457), an amine oxidase (AAEL009044), Carboxy/choline esterase (AAEL002385), 19 Cytochrome P450, and a glucosyl/glucuronosyl transferase (AAEL003099). In the control at 7 dpi, we observed six cytochromes and a glutathione transferase (AAEL007964) that were significantly expressed between the Key West and Orlando Controls fed uninfected blood (Table 1B). Nineteen Cytochrome P450 included, CYP6CB1 (AAEL009018), CYP9M10 (AAEL009125), and P450s of the CYP9J subfamily such as CYP9J10 (AAEL006798) and CYP9J28 (AAEL014617), from which several members were shown to contribute to deltamethrin metabolism [31,32,58,59]. The glucosyl/glucuronosyl transferases (AAEL003099) were reported as differentially expressed in pyrethroid resistant populations relative to the susceptible strain [32,59].

#### 3.1.6. DE Transcripts Likely Related to Permethrin resistance in Response to ZIKV Infection

Except detoxification enzymes, many other enzymes related to insecticide resistance have been reported. We analyzed the DE transcripts possible related to permethrin resistance in response to ZIKV infection. Most of the fifty-five DE transcripts likely related to permethrin resistance were upregulated in response to ZIKV 7 dpi in the Key West strain compared with the Orlando strain, but only one zinc finger protein (AAEL002388) was downregulated (Table S5A). These transcripts encoded one acetylcholine receptor, two adenylate cyclases, four alkaline phosphatases, one ATP-binding cassette transporter, three ATP-dependent bile acid permeases, two brain chitinase and chias, two bumetanide-sensitive Na-K-Cl cotransport proteins, two cgmp-dependent protein kinases, one glutamate decarboxylase, three glutamate receptors, one glutamate transporter, one glutamate-gated chloride channel, five GPCR related genes, two guanine nucleotide-binding proteins, two matrix metalloproteinases, one metalloproteinase, two prolylcarboxypeptidases, eight protease m1 zinc metalloproteases, two voltage-gated potassium channels, five zinc carboxypeptidases, four zinc finger proteins, and one zinc metalloprotease (Table S5A).

Nineteen DE transcripts likely related to permethrin resistance, except some detoxification enzymes, were regulated (14 upregulated and five downregulated) in response blood feeding control in the Key West strain compared with the Orlando strain (Table S5B). The voltage-gated sodium channel (AAEL006019) was only upregulated 1.4-fold, which may play an important role in the Key West *Ae. aegypti* strain. Between Key West *Ae. aegypti* infected with ZIKV and the Key West control at the 7 dpi, 14 DE transcripts possibly related to permethrin resistance were significantly regulated (six upregulated and eight downregulated) in response to ZIKV infection (Table S5C). Nonetheless, all 16 DE transcripts related to detoxification were significantly downregulated between the Orlando *Ae. aegypti* infected with ZIKV and the Orlando control at the 7 dpi (Table S5D).

#### 3.1.7. DE Transcripts Related to Cytoskeleton in Response to ZIKV Infection

A cytoskeleton with multitude of functions is present in all cells of all domains of life, including archaea, bacteria, and eukaryotes. The cytoskeleton assists the cell move in its environment and controls the movement of the cell’s interior workings. Our RNAseq study of two strains of *Ae. aegypti* following ZIKV infection at 7 dpi revealed that all 56 DE transcripts related to the cytoskeleton were upregulated in response to ZIKV 7 dpi in the Key West strain compared with the Orlando strain (Table S6A). These genes encoded four actin, one ca-activated cl channel protein, one cadherin, one calcium-binding protein, two calcium-transporting ATPases, one calmin, two calponin/transgelins, one calsyntenin-1 precursor, one coronin, one dynein heavy chain, one flagellar radial spoke protein, one gelsolin precursor, one gliotactin, three innexins, one integrin alpha-ps, one jnk interacting protein, one laminin, one leucokinins precursor, one mitogen activated protein kinase, one muscle lim protein, one myo inositol monophosphatase, one myoinositol oxygenase, nine myosins, one myosin regulatory light chain, one nuclear lamin L1 alpha, one nucleosome assembly protein, one otopetrin, one paramyosin, one pyrokinin, one talin, one testisin precursor, one titin protein, one tropomyosin invertebrate, five troponins, one unconventional myosin 95e isoform, and one vesamicol binding protein (Table S6A).

According to RNA-seq analysis, 21 cytoskeletons related to DE transcripts were significantly regulated (12 upregulated and nine downregulated) in response to blood feeding control in the Key West strain compared with the Orlando strain (Table S6B). Nineteen DE cytoskeleton transcripts were regulated (nine upregulated and 10 downregulated) in the ZIKV infected group of the Key West strain at the 7 dpi (Table S6C). However, all six DE transcripts related to the cytoskeleton were significantly downregulated in the ZIKV infected group of the Orlando strain at the 7 dpi (Table S6D).

We found that most genes were downregulated in the *Ae. aegypti* Orlando susceptible strain at 7 dpi following ZIKV infection. In contract, most genes were upregulated in the *Ae. aegypti* Key West permethrin resistant strain (Tables S3–S6). Compared with the *Ae. aegypti* Orlando susceptible strain at 7 dpi following ZIKV infection, *Ae. aegypti* Key West permethrin resistant strain showed a global upregulation of endogenous genes, many of which encode proteins specifically involved in immunity, detoxification, pesticide resistance, and cytoskeleton movement related genes.

## 4. Discussion

Arbovirus–mosquito interactions alter global gene expression in *Ae. aegypti* and other mosquitoes [39,40,43,48,60,61]. Although Zika infection had been reported to change transcript levels in *Ae. aegypti* [43], the mechanism(s) of insecticide resistance in *Ae. aegypti*-ZIKV remains unknown. Since insecticide-resistance monitoring is the key to controlling arboviruses, we need to improve our understanding of mosquito–virus interactions in both resistant and susceptible strains to facilitate surveillance and monitoring of Zika vector populations under control.

Many reports have been shown that the upregulated genes contained multiple detoxification genes and several immune-related genes in insecticide resistant mosquitoes, including *Ae. aegypti*, *Anopheles gambiae*, *An. sinesis*, *An. stephensi*, and *Culex quinquefasciatus* [23,31,33,62,63,64,65]. To the best of our knowledge, this is one of the first documentations showing an association between insecticide resistance and altered mosquito–arbovirus interactions. However, we are unable to rule out the possibility that inherent genetic differences between the two strains of *Ae. aegypti*, in part, contribute to differences in ZIKV infection. Although the mechanism(s) responsible for altered interactions between mosquitoes and pathogens is not fully understood, changes in oxidative stress and vector immunity have been proposed as potential sources [66]. Our observed results are consistent with Alout et al. 2013 [67] showing higher *Plasmodium falciparum* prevalence at both the oocyst and sporozoite stages, in *Anopheles gambiae* s.s. resistant to pyrethroids and DDT than in a susceptible strain. In contrast, insecticide-resistant (organophosphate) *Culex quinquefasciatus* mosquitoes were less capable of transmitting the filarial parasite *Wuchereria bancrofti* than insecticide-susceptible conspecifics, mediated by disrupted development of the parasite [68,69]. It is likely that the biological processes in response to mosquito infection of arboviruses differs from that of parasites such as filarial worms and *Plasmodium*. Regardless, taken together, these observations suggest a connection between insecticide resistance and altered physiology that translates to changes in interactions between mosquitoes and the disease agents they transmit.

To obtain a global view of changes in gene expression between *Ae. aegypti* Key West permethrin resistant strain and *Ae. aegypti* Orlando susceptible strains, we analyzed RNA-seq data and identified at least 23 detoxification enzymes linked to insecticide resistance that were significantly upregulated in response to ZIKV infection [31,32]. Our current study showed that the *Ae. aegypti* Key West permethrin resistant strain and the *Ae. aegypti* Orlando susceptible strain differentially altered their gene expression in response to ZIKV infection.

To survive in a world full of pathogens, insects have developed a powerful defense mechanism that recognizes and removes microbial threats [70]. Insects depend on innate immunity for their survival. The immune system accommodates host colonization by the virus, maintains virus–host homeostasis and defends against pathogens. Viral infections are detected by innate antiviral responses [71]. Pathogen receptors in the innate immune system play a role in the detection of viral nucleic acids in different ways [71]. Toll-like receptors detected viral DNA or RNA in endosomal compartments in immune cells [45,72], while retinoic acid inducible gene-I-like receptors recognized viral RNA in the cytoplasm and DNA sensors detected cytoplasmic viral DNA [71]. The Toll pathways have previously been shown to suppress arbovirus infection in *Ae. aegypti* midgut tissue [45]. Peptidoglycan recognition proteins, conserved from insects to mammals, are pattern recognition molecules that recognize microbes and their unique cell wall component, peptidoglycan [73]. Our transcriptomic study revealed that five Toll-like receptors and three peptidoglycan recognition proteins were significantly upregulated in the Key West permethrin resistant strain *Ae. aegypti* at 7 dpi following ZIKV infection compared with the Orlando susceptible *Ae. aegypti* (Table S3A,B). Clip-domain serine proteases are the essential components of extracellular signaling cascades in various biological processes and function in developmental processes and innate immune responses [74,75]. Twelve Clip-domain serine proteases were upregulated between the Key West permethrin resistant and the Orlando susceptible strains of *Ae. aegypti*. CLIP proteases are found in insect hemolymph and participate in cascade pathways that activate prophenoloxidase in the melanization response and synthesis of antimicrobial peptides [74], including immune signaling in *Ae. aegypti* [76,77].

Other immune related enzymes, such as cecropin antimicrobial peptide, Class B scavenger receptor, defensin antimicrobial peptide, fibrinogen and fibronectin, and leucine-rich immune proteins were also upregulated between the Key West permethrin resistant strain *Ae. aegypti* and the Orlando susceptible *Ae. aegypti*. Cysteine-rich venom proteins, found in the fluids of animal venoms, inhibit both smooth muscle contraction and cyclic nucleotide-gated ion channels [78]. Previous studies displayed that cysteine-rich venom proteins were changed in yellow fever and ZIKV-infected mosquitoes and silencing the gene led to an increase in replication of dengue viruses, which indicated their possible importance in replication of these viruses [43,79]. The current study showed that cysteine-rich venom proteins (AAEL005098) were upregulated 2.71 log2 fold change in the Key West strain ZIKV compared with Key West control, and upregulated 5.77 log2 fold change when compared with the Orlando strain *Ae. aegypti* in response to ZIKV. Further studies may need to demonstrate the role of cysteine-rich venom proteins play in response to Zika infection. These data indicated the permethrin resistant Key West *Ae. aegypti* mosquitoes altered immune system in response to ZIKV infection, differently from the susceptible Orlando *Ae. aegypti* strain.

The activation of multiple signaling pathways following virus infection, the detoxification genes implicated in the establishment of the antiviral state, and the strategies used by viruses and their specific viral products to antagonize and evade the host antiviral response. Recent studies have utilized [31] deep targeted DNA sequencing for identification of increases in gene copy number in the genome associated with pyrethroid resistance in populations of *Ae. aegypti* and subsequently identified novel genomic resistance markers potentially associated with their cis-regulation and modifications of their protein structure confirmation [31,32]. The current RNA-seq study also confirmed 23 over expression of detoxification enzymes associated with insecticide resistance in Key West *Ae. aegypti* in response to ZIKV 7 dpi compared with Orlando *Ae. aegypti* susceptible strain. CYP6CB1-like AAEL009018, considerably favor the binding of an HNF-3 element and overexpression of this gene in resistant populations has frequently been associated with the regulation of drug-metabolizing P450s [31,49]. CYP9M10, AAEL009125, was not only demonstrated in the *Ae. aegypti* mosquito but also reported in the resistant strain of *Culex quinquefasciatus* [31,80,81]. The current study also confirmed that detoxification enzymes, such as carboxy/choline esterase and glucosyl/glucuronosyl transferases, were associated with resistance mosquitoes in response to ZIKV infection [31,32,82,83]. The mechanisms for regulation of detoxification enzymes in response to ZIKV and their relevance to insecticide resistance are unclear. It has been proposed that regulation in some metabolic detoxification genes may result from responses to various endogenous and exogenous compounds, or to pathophysiological signals [33,63,64,84,85].

The actin and microtubule cytoskeleton play important roles in the life cycle of viruses. Viruses succeed as intracellular parasites and interact with the actin cytoskeleton at various stages of the host cell throughout their life cycles to facilitate the infection process [86,87]. Many animal viruses interact with cytoskeleton elements inside infected cells at different stages of replication and cytoskeleton involvement in virus budding [88]. The microfilament signal pathway is involved in DENV infection through regulation of actin reorganization in EAhy926 cells [89]. Viral interaction with the host microtubule (MT) cytoskeleton is critical to infection by many viruses, with modifying MT dynamics and functions that affect processes beyond virion transport [90]. Myosin protein enforced track selection on the microtubule and actin networks in vitro, depending on the active transport of diverse intracellular cargo on the ubiquitous actin and microtubule networks [91]. Some studies showed that the manipulation of host actin cytoskeleton is essential for viral pathogens to invade the host cells [92]. Our current data show that 56 DE transcripts related to cytoskeleton, including four actin and 10 myosin proteins, were significantly upregulated in the Zika infected Key West strain compared with the Orlando strain *Ae. aegypti* 7-day post infection. The overexpression of actin cytoskeleton genes in the permethrin resistance strain of *Ae. aegypti* might be associated with higher viral loads later during the infection process, although the precise functional importance of these interactions and their roles in pathogenesis remain largely unresolved.

Our observations provide an overview of gene expression associated with metabolic detoxification among permethrin resistant and susceptible populations of *Ae. aegypti*, including antiviral responses following ingestion of ZIKV. Our understanding of host–virus interactions in mosquito systems combining traditional genetic and biochemical approaches with ”omics” based approaches in both laboratory and natural environmental studies is key to improving the surveillance and monitoring of Zika vector populations under control. One of the limitations of this approach is that it falls short of providing an in-depth analysis of any one specific mechanism, or collection of mechanisms. Rather, our broad approach is aimed at providing a global view to identify candidate genes and functional categories for subsequent studies using other methods (e.g., reverse genetics) that target candidate genes for elucidating molecular mechanisms of insecticide resistance and the development of novel molecular mechanisms to circumvent resistance.

## Figures and Tables

**Table 1:** Transcription profiles of detoxification enzymes associated with permethrin resistance. (A) Genes related to detoxification significant upregulated in the Zika infection in the permethrin resistant (KW) strain compared with susceptible (OR) strain *Aedes aegypti* 7-days post infection. (B) Detoxification related gene significant upregulated/downregulated in the Control in the permethrin resistant (KW) strain compared with the susceptible (OR) strain *Aedes aegypti* 7-days post injection.

Gene ID	logFC	p-adj	Gene Description	Gene Name	Publications
**A**					
AAEL012457	2.2934	3.09 × 10^−10^	alcohol dehydrogenase		Faucon et al., 2015 [32]
AAEL009044	2.5174	4.04 × 10^−11^	amine oxidase		Faucon et al., 2015 [32]
AAEL002385	2.0902	2.33 × 10^−3^	Carboxy/choline esterase	CCEAE3B	Dusfour, 2015 [49]
AAEL001960	2.1283	7.7 × 10^−7^	cytochrome P450	CYP6M9	Faucon et al., 2015 [32]
AAEL002031	1.837	1.37 × 10^−19^	cytochrome P450	CYP12F7	Faucon et al., 2015 [32]
AAEL006798	2.8656	8.05 × 10^−3^	cytochrome P450	CYPJ10	Faucon et al., 2017 [31]
AAEL006805	2.6907	6.0 × 10^−18^	cytochrome P450	CYP9J2	Faucon et al., 2015 [32]
AAEL006815	2.4201	6.0 × 10^−17^	cytochrome P450	CYP9J16	Faucon et al., [31,32]
AAEL007473	2.1909	5.5 × 10^−15^	cytochrome P450	CYP6AH1	Faucon et al., 2015 [32]
AAEL009018	2.5349	4.5 × 10^−8^	cytochrome P450	CYP6CB1	Faucon et al., 2015 [32]
AAEL009123	2.4747	7.8 × 10^−9^	cytochrome P450	CYP6Z6	Faucon et al., 2017 [31]
AAEL009125	2.6551	7.0 × 10^−13^	cytochrome P450	CYP6M10	Faucon et al., 2017 [31]
AAEL009129	2.4377	2.6 × 10^−34^	cytochrome P450	CYP6Z9	Faucon et al., 2017 [31]
AAEL014603	2.4789	2.24 × 10^−3^	cytochrome P450	CYP9J30	Faucon et al., [31,32]
AAEL014607	2.1961	4.6 × 10^−4^	cytochrome P450	CYP9J?	Faucon et al., 2015 [32]
AAEL014608	1.9517	8.2 × 10^−3^	cytochrome P450	CYP9J?	Faucon et al., 2017 [31]
AAEL014609	2.4159	1.1 × 10^−12^	cytochrome P450	CYP9J26	Faucon et al., [31,32]
AAEL014614	3.9363	4.1 × 10^−8^	cytochrome P450	CYP9J?	Faucon et al., 2015 [32]
AAEL014617	2.3975	5.7 × 10^−4^	cytochrome P450	CYP9J28	Faucon et al., 2015 [32]
AAEL014893	2.2053	2.1 × 10^−8^	cytochrome P450	CYP6BB2	Faucon et al., 2015 [32]
AAEL015663	4.0198	9.0 × 10^−6^	cytochrome P450	CYP25?	Faucon et al., 2015 [32]
AAEL017297	3.2113	1.7 × 10^−6^	cytochrome P450	CYP6M9	Faucon et al., [31,32]
AAEL003099	3.0651	1.4 × 10^−4^	glucosyl/glucuronosyl transferases		Faucon et al., 2015 [32]
**B**					
AAEL001312	2.0651	2.5 × 10^−4^	cytochrome P450	CYP9M6	Faucon et al., 2017 [31]
AAEL006798	3.6029	5.1 × 10^−4^	cytochrome P450	CYP9J10	Faucon et al., 2015 [32]
AAEL006811	2.5971	2.37 × 10^−3^	cytochrome P450	CYP9J8	Faucon et al., [31,32]
AAEL014606	1.9372	1.49 × 10^−6^	cytochrome P450	CYPJ7	Faucon et al., 2015 [32]
AAEL014617	2.2615	3.35 × 10^−3^	cytochrome P450	CYPJ28	Faucon et al., 2015 [32]
AAEL014891	−2.8122	3.8 × 10^−4^	cytochrome P450	CYP6P12	Faucon et al., 2017 [31]
AAEL007947	2.5390	8.31 × 10^−32^	glutathione transferase	GSTE	Faucon et al., 2017 [31]

## Supplementary Materials

The following are available online at http://www.mdpi.com/1999-4915/10/9/470/s1, Figure S1: GO analyses for RNA-seq data. Table S1: Primers for validation of the expression of transcripts between two strains of *Aedes aegypti*. Table S2. Summary of RNA-seq analysis based on the *Aedes aegypti* transcriptomes. Tables S3–S6: Related gene significant upregulated/downregulated.
