# Supplementary material for: The Effect of Permethrin Resistance on Aedes aegypti Transcriptome Following Ingestion of Zika Virus Infected Blood
Source: Viruses. 2018 Sep 1;10(9):470. doi: 10.3390/v10090470 (PMC6165428; doi:10.3390/v10090470)
Supplement: Supplementary file 1 [file viruses-10-00470-s001.zip › 08092018-Supplementary S1-TableS1.docx]

**Supplementary S1:**

**Table S1**. Primers for validation of the expression of transcripts between two strains of *Aedes aegypti*.
