# Supplementary material for: The Effect of Permethrin Resistance on Aedes aegypti Transcriptome Following Ingestion of Zika Virus Infected Blood
Source: Viruses. 2018 Sep 1;10(9):470. doi: 10.3390/v10090470 (PMC6165428; doi:10.3390/v10090470)
Supplement: Supplementary file 1 [file viruses-10-00470-s001.zip › 08092018-Supplementary S2-TableS2.docx]

**Supplementary S2:**

**Table S2**. Summary of RNA-seq analysis based on the *Aedes* *aegypti* transcriptomes (18,840 genes).

* AB1, 2, 3-KW-Cont: three replicates of Key West *Ae. aegypti*, 12-hour after blood only injection.

* ab1, 2, 3-KW-ZikV: three replicates of Key West *Ae. aegypti*, 12-hour after ZIKV injection.

* HI1, 2, 3-OR-Cont: three replicates of Orlando *Ae. aegypti*, 12-hour after blood only injection.

* hi1, 2, 3-OR-ZikV: three replicates of Orlando *Ae. aegypti*, 12-hour after ZIKV injection.

* FF1, 2, 3-KW-Cont: three replicates of Key West *Ae. aegypti*, 7-day after blood only injection.

* ff1, 2, 3-KW-ZikV: three replicates of Key West *Ae. aegypti*, 7-day after ZIKV injection.

* M1, 2, 3-OR-Cont: three replicates of Orlando *Ae. aegypti*, 7-day after blood only injection.

* ff1, 2, 3-OR-ZikV: three replicates of Orlando *Ae. aegypti*, 7-day after ZIKV injection.
