# Supplementary material for: The Effect of Permethrin Resistance on Aedes aegypti Transcriptome Following Ingestion of Zika Virus Infected Blood
Source: Viruses. 2018 Sep 1;10(9):470. doi: 10.3390/v10090470 (PMC6165428; doi:10.3390/v10090470)
Supplement: Supplementary file 1 [file viruses-10-00470-s001.zip › 08092018-Supplementary S4-Go-Analyses-Figure S1.docx]

**Supplementary S3---****Figure S1.** **GO analyses for RNA-seq data.**

**Figure S1A**. 12 hours post injection KW-Control compared with OR-Control (**Figure 3A**).

| **Functional categories** |  | **Gene No.** | **Percentage** |
| --- | --- | --- | --- |
| GO:0002376 | Immune system process | 0 | 0% |
| GO:0050896 | Response to stimulus | 2 | 5.71% |
| GO:0032502 | Developmental process | 0 | 0% |
| GO:0009987 | Cellular process | 3 | 8.57% |
| GO:0004871 | Signal transducer activity | 0 | 0% |
| GO:0065007 | Biological regulation | 0 | 0% |
| GO:0050789 | Regulation of biological regulation | 0 | 0% |
| GO:0009055 | Electron carrier activity | 0 | 0% |
| GO:0005215 | Transporter activity | 4 | 11.43% |
| GO:0003824 | Catalytic activity | 6 | 17.14% |
| GO:0008152 | Metabolic process | 1 | 2.86% |
| GO:0005488 | Binding | 7 | 20% |
| GO:0005198 | Structural molecular activity | 0 | 0% |
|  |  | 23 |  |
|  | Unknown | 12 | 34.29% |
| **Figure S1A up** | Total | 35 |  |

| **Functional categories** |  | **Gene No.** | **Percentage** |
| --- | --- | --- | --- |
| GO:0002376 | Immune system process | 0 | 0% |
| GO:0050896 | Response to stimulus | 3 | 5.45% |
| GO:0032502 | Developmental process | 0 | 0% |
| GO:0009987 | Cellular process | 8 | 14.55% |
| GO:0004871 | Signal transducer activity | 0 | 0% |
| GO:0065007 | Biological regulation | 0 | 0% |
| GO:0050789 | Regulation of biological regulation | 0 | 0% |
| GO:0009055 | Electron carrier activity | 0 | 0% |
| GO:0005215 | Transporter activity | 0 | 0% |
| GO:0003824 | Catalytic activity | 7 | 12.73% |
| GO:0008152 | Metabolic process | 0 | 0% |
| GO:0005488 | Binding | 14 | 25.45% |
| GO:0005198 | Structural molecular activity | 0 | 0% |
|  |  | 32 |  |
|  | Unknown | 23 | 41.82% |
| **Figure S1A down** | Total | 55 |  |

**Figure S1B**. 12 hours post infection, KW-ZIKV compared with OR-ZIKV (**Figure 3B**).

| **Functional categories** |  | **Gene No.** | **Percentage** |
| --- | --- | --- | --- |
| GO:0002376 | Immune system process | 0 | 0% |
| GO:0050896 | Response to stimulus | 1 | 3.57% |
| GO:0032502 | Developmental process | 0 | 0% |
| GO:0009987 | Cellular process | 0 | 0% |
| GO:0004871 | Signal transducer activity | 0 | 0% |
| GO:0065007 | Biological regulation | 0 | 0% |
| GO:0050789 | Regulation of biological regulation | 0 | 0% |
| GO:0009055 | Electron carrier activity | 0 | 0% |
| GO:0005215 | Transporter activity | 5 | 17.86% |
| GO:0003824 | Catalytic activity | 5 | 17.86% |
| GO:0008152 | Metabolic process | 1 | 3.57% |
| GO:0005488 | Binding | 4 | 14.29% |
| GO:0005198 | Structural molecular activity | 0 | 0% |
|  |  | 16 |  |
|  | Unknown | 12 | 42.86% |
| **Figure S1B up** | Total | 28 |  |

| **Functional categories** |  | **Gene No.** | **Percentage** |
| --- | --- | --- | --- |
| GO:0002376 | Immune system process | 0 | 0% |
| GO:0050896 | Response to stimulus | 0 | 0% |
| GO:0032502 | Developmental process | 0 | 0% |
| GO:0009987 | Cellular process | 4 | 8.16% |
| GO:0004871 | Signal transducer activity | 0 | 0% |
| GO:0065007 | Biological regulation | 0 | 0% |
| GO:0050789 | Regulation of biological regulation | 0 | 0% |
| GO:0009055 | Electron carrier activity | 1 | 2.04% |
| GO:0005215 | Transporter activity | 3 | 6.12% |
| GO:0003824 | Catalytic activity | 6 | 12.24% |
| GO:0008152 | Metabolic process | 1 | 2.04% |
| GO:0005488 | Binding | 10 | 20.41% |
| GO:0005198 | Structural molecular activity | 0 | 0% |
|  |  | 25 |  |
|  | Unknown | 24 | 48.98% |
| **Figure S1B down** | Total | 49 |  |

**Figure S1C**. 7 dpi, KW-ZIKV compared with KW-Control (**Figure 3C**).

| **Functional categories** |  | **Gene No.** | **Percentage** |
| --- | --- | --- | --- |
| GO:0002376 | Immune system process | 1 | 0.19% |
| GO:0050896 | Response to stimulus | 38 | 7.04% |
| GO:0032502 | Developmental process | 1 | 0.19% |
| GO:0009987 | Cellular process | 77 | 14.25% |
| GO:0004871 | Signal transducer activity | 2 | 0.37% |
| GO:0065007 | Biological regulation | 0 | 0% |
| GO:0050789 | Regulation of biological regulation | 0 | 0% |
| GO:0009055 | Electron carrier activity | 1 | 0.19% |
| GO:0005215 | Transporter activity | 22 | 4.07% |
| GO:0003824 | Catalytic activity | 85 | 15.74% |
| GO:0008152 | Metabolic process | 4 | 0.74% |
| GO:0005488 | Binding | 119 | 22.04% |
| GO:0005198 | Structural molecular activity | 1 | 0.19% |
|  |  | 351 |  |
|  | Unknown | 189 | 35.00% |
| **Figure S1C up** | Total | 540 |  |

| **Functional categories** |  | **Gene No.** | **Percentage** |
| --- | --- | --- | --- |
| GO:0002376 | Immune system process | 3 | 7.65% |
| GO:0050896 | Response to stimulus | 32 | 8.16% |
| GO:0032502 | Developmental process | 1 | 0.25% |
| GO:0009987 | Cellular process | 62 | 15.82% |
| GO:0004871 | Signal transducer activity | 3 | 7.65% |
| GO:0065007 | Biological regulation | 0 | 0% |
| GO:0050789 | Regulation of biological regulation | 0 | 0% |
| GO:0009055 | Electron carrier activity | 1 | 0.26% |
| GO:0005215 | Transporter activity | 19 | 4.85% |
| GO:0003824 | Catalytic activity | 55 | 14.03% |
| GO:0008152 | Metabolic process | 4 | 1.02% |
| GO:0005488 | Binding | 63 | 16.07% |
| GO:0005198 | Structural molecular activity | 1 | 0.26% |
|  |  | 244 |  |
|  | Unknown | 148 | 37.76% |
| **Figure S1C down** | Total | 392 |  |

**Figure S1D**. 7 dpi, OR-ZIKV compared with OR-Control (**Figure 3D**).

| **Functional categories** |  | **Gene No.** | **Percentage** |
| --- | --- | --- | --- |
| GO:0002376 | Immune system process | 0 | 0% |
| GO:0050896 | Response to stimulus | 1 | 3.85% |
| GO:0032502 | Developmental process | 0 | 0% |
| GO:0009987 | Cellular process | 5 | 19.23% |
| GO:0004871 | Signal transducer activity | 0 | 0% |
| GO:0065007 | Biological regulation | 0 | 0% |
| GO:0050789 | Regulation of biological regulation | 0 | 0% |
| GO:0009055 | Electron carrier activity | 0 | 0% |
| GO:0005215 | Transporter activity | 2 | 7.69% |
| GO:0003824 | Catalytic activity | 5 | 19.23% |
| GO:0008152 | Metabolic process | 0 | 0% |
| GO:0005488 | Binding | 2 | 7.69% |
| GO:0005198 | Structural molecular activity | 0 | 0% |
|  |  | 15 |  |
|  | Unknown | 11 | 42.31% |
| **Figure S1D up** | Total | 26 |  |

| **Functional categories** |  | **Gene No.** | **Percentage** |
| --- | --- | --- | --- |
| GO:0002376 | Immune system process | 1 | 3.06% |
| GO:0050896 | Response to stimulus | 14 | 4.28% |
| GO:0032502 | Developmental process | 1 | 3.06% |
| GO:0009987 | Cellular process | 49 | 14.98% |
| GO:0004871 | Signal transducer activity | 1 | 0.31% |
| GO:0065007 | Biological regulation | 1 | 0.31% |
| GO:0050789 | Regulation of biological regulation | 0 | 0% |
| GO:0009055 | Electron carrier activity | 1 | 0.31% |
| GO:0005215 | Transporter activity | 18 | 5.50% |
| GO:0003824 | Catalytic activity | 59 | 18.04% |
| GO:0008152 | Metabolic process | 3 | 0.92% |
| GO:0005488 | Binding | 56 | 17.13% |
| GO:0005198 | Structural molecular activity | 4 | 1.00% |
|  |  | 208 |  |
|  | Unknown | 119 | 36.39% |
| **Figure S1D down** | Total | 327 |  |

**Figure S1E**. 7 dpi, KW-Control compared with OR-Control; **F**. 7 dpi, KW-ZIKV compared with OR-ZIKV (**Figure 3E**)**.**

| **Functional categories** |  | **Gene No.** | **Percentage** |
| --- | --- | --- | --- |
| GO:0002376 | Immune system process | 3 | 1.03% |
| GO:0050896 | Response to stimulus | 27 | 9.28% |
| GO:0032502 | Developmental process | 0 | 0% |
| GO:0009987 | Cellular process | 40 | 13.75% |
| GO:0004871 | Signal transducer activity | 3 | 1.03% |
| GO:0065007 | Biological regulation | 1 | 0.34% |
| GO:0050789 | Regulation of biological regulation | 0 | 0% |
| GO:0009055 | Electron carrier activity | 1 | 0.34% |
| GO:0005215 | Transporter activity | 17 | 5.84% |
| GO:0003824 | Catalytic activity | 43 | 14.78% |
| GO:0008152 | Metabolic process | 3 | 1.03% |
| GO:0005488 | Binding | 45 | 15.46% |
| GO:0005198 | Structural molecular activity | 0 | 0% |
|  |  | 183 |  |
|  | Unknown | 108 | 37.11% |
| **Figure S1E up** | Total | 291 |  |

| **Functional categories** |  | **Gene No.** | **Percentage** |
| --- | --- | --- | --- |
| GO:0002376 | Immune system process | 0 | 0% |
| GO:0050896 | Response to stimulus | 23 | 6.76% |
| GO:0032502 | Developmental process | 0 | 0% |
| GO:0009987 | Cellular process | 49 | 14.41% |
| GO:0004871 | Signal transducer activity | 0 | 0% |
| GO:0065007 | Biological regulation | 0 | 0% |
| GO:0050789 | Regulation of biological regulation | 0 | 0% |
| GO:0009055 | Electron carrier activity | 2 | 0.59% |
| GO:0005215 | Transporter activity | 13 | 3.82% |
| GO:0003824 | Catalytic activity | 45 | 13.24% |
| GO:0008152 | Metabolic process | 1 | 0.29% |
| GO:0005488 | Binding | 81 | 23.82% |
| GO:0005198 | Structural molecular activity | 2 | 0.59% |
|  |  | 216 |  |
|  | Unknown | 124 | 36.47% |
| **Figure S1E down** | Total | 340 |  |

**Figure S1F**. 7 dpi, KW-ZIKV compared with OR-ZIKV (**Figure 3F**).

| **Functional categories** |  | **Gene No.** | **Percentage** |
| --- | --- | --- | --- |
| GO:0002376 | Immune system process | 9 | 0.46% |
| GO:0050896 | Response to stimulus | 99 | 5.11% |
| GO:0032502 | Developmental process | 8 | 0.41% |
| GO:0009987 | Cellular process | 255 | 13.17% |
| GO:0004871 | Signal transducer activity | 15 | 0.77% |
| GO:0065007 | Biological regulation | 4 | 0.21% |
| GO:0050789 | Regulation of biological regulation | 0 | 0% |
| GO:0009055 | Electron carrier activity | 5 | 0.26% |
| GO:0005215 | Transporter activity | 129 | 6.66% |
| GO:0003824 | Catalytic activity | 384 | 19.83% |
| GO:0008152 | Metabolic process | 18 | 0.93% |
| GO:0005488 | Binding | 290 | 14.98% |
| GO:0005198 | Structural molecular activity | 12 | 0.62% |
|  |  | 1228 |  |
|  | Unknown | 708 | 36.57% |
| **Figure S1F up** | Total | 1936 |  |

| **Functional categories** |  | **Gene No.** | **Percentage** |
| --- | --- | --- | --- |
| GO:0002376 | Immune system process | 0 | 0% |
| GO:0050896 | Response to stimulus | 24 | 4.59% |
| GO:0032502 | Developmental process | 0 | 0% |
| GO:0009987 | Cellular process | 115 | 21.99% |
| GO:0004871 | Signal transducer activity | 1 | 0.19% |
| GO:0065007 | Biological regulation | 0 | 0% |
| GO:0050789 | Regulation of biological regulation | 0 | 0% |
| GO:0009055 | Electron carrier activity | 0 | 0% |
| GO:0005215 | Transporter activity | 7 | 1.34% |
| GO:0003824 | Catalytic activity | 63 | 12.05% |
| GO:0008152 | Metabolic process | 0 | 0% |
| GO:0005488 | Binding | 146 | 27.92% |
| GO:0005198 | Structural molecular activity | 1 | 0.19% |
|  |  | 357 |  |
|  | Unknown | 166 | 31.74% |
| **Figure S1F down** | Total | 523 |  |
