# Supplementary material for: The Effect of Permethrin Resistance on Aedes aegypti Transcriptome Following Ingestion of Zika Virus Infected Blood
Source: Viruses. 2018 Sep 1;10(9):470. doi: 10.3390/v10090470 (PMC6165428; doi:10.3390/v10090470)
Supplement: Supplementary file 1 [file viruses-10-00470-s001.zip › 08292018-Supplementary S3-Table 3A-6D-GeneExpression-2FOLD.docx]

**Supplementary Table S3-S6:** Related genes significant upregulated/downregulated (p-adj ≤0.01; log2 fold change > + 2.0)**.**

**Table S3A**. Immunity related genes significantly upregulated in the Zika infection in Key West strain compared with Orlando strain *Aedes aegypti* 7-days post infection.

**Table S3B**. Immunity related genes significantly upregulated/downregulated in the Control in Key West strain compared with Orlando strain *Aedes aegypti* 7-days post injection.

**Table S3C**. Immunity related genes significantly dysregulated in the Key West strain *Aedes aegypti* 7-days post infection with ZIKV compared with Control in Key West strain.

**Table S3D**. Immunity related genes significantly dysregulated in the Orlando strain *Aedes aegypti* 7-days post infection with ZIKV compared with Control in Orlando strain.

***3.1.4 DE transcripts related to immunity in response to ZIKV infection***

Analysis and comparison of mRNA expression proﬁles of *Ae. aegypti* mosquitoes at different two strains following blood injection revealed that in total 360 transcripts had changes of 2-fold or more in either direction (p-adj ≤0.01; log2 fold change > + 2.0). According to RNA-seq analysis, 18 DE transcripts related to immunity were significantly regulated (13-upregulated and 5-downregulated) in response to blood feeding controls 7-days post infection in the Key West strain compared with Orlando strain control (**Table S3B**). The transcripts related to innate immunity encoded one Class C Scavenger Receptor, four Clip-domain serine proteases Family A and B, one C-type lectin, one defensin anti-microbial peptide, one expressed protein, one fibrinogen and fibronectin, one follistatin, one inosine-uridine preferring nucleoside hydrolase, two ionotropic glutamate receptors, one lachesin, one M protein, one prophenoloxidase (AAEL013496), one shoc2, and one venom allergen (**Table S3B**). Six genes were up/downregulated more than 4-fold, including defensin anti-microbial peptides (AAEL003832), lachesin (AAEL000576), shoc2 (AAEL009928) and venom allergen (AAEL013406).

We correspondingly compared the Key West *Ae. aegypti* infected with ZIKV with the Key West control at the 7-dpi and revealed that in total 318 transcripts had changes of 2-fold or more in either direction. Fifteen DE transcripts related to immunity were significantly dysregulated more than two-fold (7 upregulated and 8-downregulated, **Table S3C**). These transcripts encoded two Class C Scavenger Receptors, two Clip-domain serine proteases family B, two C-type lectins, one cysteine-rich venom protein (AAEL005098, 2.71 log2 fold change), one Gram-negative binding Protein, one lachesin, three leucine-rich transmembrane proteins, a shoc2, one venom allergen, and one Wnt10a protein (**Table S3C**).

Analysis and comparison of mRNA expression proﬁles of *Ae. aegypti* mosquitoes at the Orlando strain following ZIKV infection at 7 dpi revealed that in total 128 transcripts had changes of 2-fold or more, only one was upregulated. All 14 DE transcripts related to immunity were significantly downregulated between Orlando *Ae. aegypti* infected with ZIKV and the Orlando control at the 7 dpi (**Table S3D**). These transcripts encoded six Clip-domain serine proteases family B, one C-type lectin, five leucine-rich immune proteins, one Trypsin 3A1 precursor and one tyrosine kinase receptor (**Table S3D**). Both the Key West *Ae. aegypti* and Orlando strains infected with ZIKV at the 7dpi shown regulated with Clip-domain serine protease family B, C-type lectin, and some leucine-rich proteins.

**Table S4A**. Detoxification related genes significantly upregulated in the Zika infection treatment group of Key West strain compared with Orlando strain *Aedes aegypti* 7-days post infection.

**Table S4B**. Detoxification related genes significantly upregulated/downregulated in the Control in Key West strain compared with Orlando strain *Aedes aegypti* 7-days post injection.

**Table S4C**. Detoxification related genes significantly dysregulated in the Key West strain *Aedes aegypti* 7 pdi with ZIKV compared with Control in Key West strain.

**Table S4D**. Detoxification related genes significantly dysregulated in the Orlando strain *Aedes aegypti* 7 pdi with ZIKV compared with Control in Orlando strain.

***3.1.5 DE transcripts related to detoxification in response to ZIKV infection***

According to RNA-seq analysis, fifteen DE transcripts related to detoxification were regulated (6-upregulated and 9-downregulated) in response blood feeding control in the Key West strain compared with Orlando strain (**Table S4B**). These transcripts encoded one aldehyde dehydrogenenase, one cytochrome b5, eight cytochrome P450s, one glucosyl/glucuronosyl transferases, two glutathione transferases, and one short-chain dehydrogenase. Among the cytochrome P450 (AAEL014617, AAEL006811) and glutathione transferase (AAEL007964) was reported associated with insecticide resistance (Faucon et al. 2015, Faucon et al. 2017) and upregulated more than two-fold in the Key West strain compared with Orlando strain.

In addition, we also compared Key West *Ae. aegypti* infected with ZIKV with the Key West control at the 7 dpi, nineteen DE transcripts related to detoxification were significantly dysregulated (11 upregulated and 8-downregulated, **Table S4C**). These detoxification genes encoded one aldehyde dehydrogenase, one amine oxidase, one cytochrome b5, eight cytochrome P450 (AAEL014617 associated with resistance), one Double Oxidase: Two Peroxidase domains, one glucose dehydrogenase, two glucosyl/glucuronosyl transferases, one glutathione transferase, one multicopper oxidase, one prophenoloxidase and one sterol desaturase (**Supplementary S3-Table S4C**).

Nevertheless, all 14 DE transcripts related to detoxification were significantly dysregulated between Orlando *Ae. aegypti* infected with ZIKV and the Orlando control at the 7 dpi (**Table S4D**). These transcripts encoded one cytochrome b5, six cytochrome P450, one epoxide hydrolase, two glucosyl/glucuronosyl transferases, one glutamate semialdehyde dehydrogenase, two short-chain dehydrogenases, and one sterol desaturase (**Table S4D**). The cytochrome b5 and glucosyl/glucuronosyl transferases were significantly downregulated in the Orlando strain *Ae. aegypti* at 7 dpi infected with ZIKV, while they were significantly upregulated in the Key West strain *Ae. aegypti* at 7 dpi infected with ZIKV. However, two out of 8 cytochrome P450 were significantly upregulated in the Key West strain *Ae. aegypti* 7 dpi infected with ZIKV (**Table S4C**).

**Table S5A**. Resistance related gene (exclude detoxification enzymes) significant upregulated in the Zika infection in Key West strain compared with Orlando strain *Aedes aegypti* 7-day post infection.

**Table S5B**. Resistance related gene (exclude detoxification enzymes) significant upregulated/downregulated in the Control in Key West strain compared with Orlando strain *Aedes aegypti* 7-day post injection.

**Table S5C**. Resistance related gene (exclude detoxification enzymes) significant dysregulated in the Key West strain *Aedes aegypti* 7 pdi with ZIKV compared with Control in Key West strain.

**Table S5D**. Resistance related gene (exclude detoxification enzymes) significant dysregulated in the Orlando strain *Aedes aegypti* 7 pdi with ZIKV compared with Control in Orlando strain.

***3.1.6 DE transcripts likely related to resistance in response to ZIKV infection***

Except detoxification enzymes, many other enzymes related insecticide resistance have been reported. We analyzed the DE transcripts possibly related to permethrin resistance in response to ZIKV infection. Nineteen DE transcripts possibly related to permethrin resistance, except some detoxification enzymes, were regulated (14-upregulated and 5-downregulated) in response blood feeding control in the Key West strain compared with Orlando strain (**Table S5B**). These transcripts encoded two cdp-diacylglycerol--glycerol-3-phosphate 3-phosphatidyltransferases, two cyclic-nucleotide-gated cation channels, one glutamate receptor, five GPCR related genes, one GTP-binding protein rit, one guanine nucleotide exchange factor, one hypoxia-inducible factor, one inosine-uridine preferring nucleoside hydrolase, two ionotropic glutamate receptor, one kidney-specific Na-K-Cl cotransport protein, one potassium channel interaction protein, and one sulfonylurea receptor/ ABC transporter protease (**Table S5B**). The voltage-gated sodium channel (AAEL006019) were only upregulated 1.4-fold (see the sequencing data), which may play an important role in the Key West *Ae. aegypti* strain.

Between Key West *Ae. aegypti* infected with ZIKV and the Key West control at the 7 dpi, fourteen DE transcripts possible related to permethrin resistance were significantly dysregulated (6-upregulated and 8-downregulated) in response to ZIKV infection (**Table S5C**). These genes encoded one adenylate cyclase, one calsenilin, three cyclic-nucleotide-gated cation channel, one glucose dehydrogenase, one glucose-methanol-choline oxidoreductase, one glutamate receptor, four GPCR related genes, one GTP-binding protein, and one protease m1 zinc metalloprotease (**Table S5C**).

Nonetheless, all sixteen DE transcripts related to detoxification were significantly dysregulated between Orlando *Ae. aegypti* infected with ZIKV and the Orlando control at the 7 dpi (**Table S5D**). These transcripts encoded one adenylate cyclase, two alkaline phosphatase, one AMP dependent coa ligase, one brain chitinase and chia, two bumetanide-sensitive Na-K-Cl cotransport protein, one glutamate decarboxylase, one glutamate receptor, one glutamate semialdehyde dehydrogenase, one inward-rectifying potassium channel, one matrix metalloproteinase, one metalloproteinase, one protein serine/threonine kinase, and one zinc carboxypeptidase (**Table S5D**).

**Table S6A**. Cytoskeleton related gene significant upregulated in the Zika infection in Key West strain compared with Orlando strain *Aedes aegypti* 7-day post infection.

**Table S6B**. Cytoskeleton related gene significant upregulated/downregulated in the Control in Key West strain compared with Orlando strain *Aedes aegypti* 7-day post injection.

**Table S6C**. Cytoskeleton related gene significant dysregulated in the Key West strain *Aedes aegypti* 7 days post infection with ZIKV compared with Control in Key West strain.

**Table S6D**. Cytoskeleton related gene significant dysregulated in the Orlando strain *Aedes aegypti* 7 days post infection with ZIKV compared with Control in Orlando strain.

***3.1.7 DE transcripts related to cytoskeleton in response to ZIKV infection***

According to RNA-seq analysis, twenty-one cytoskeletons related DE transcripts were significantly regulated (12-upregulated and 9-downregulated) in response blood feeding control in the Key West strain compared with Orlando strain (**Table S6B**). These transcripts encoded one actin, one activin receptor, two adams, one basic helix-loop-helix zip transcription factor, two cadherin, one calmodulin, one calponin/transgelin, one diaphanous, one dynactin, a follistatin, one merlin/moesin/ezrin/radixin, one myelin transcription factor 1, one nuclear lamin L1 alpha, one rolling pebbles, one sidestep protein, one troponin, two tubulin alpha chains, and one tubulin-specific chaperone (**Table S6B**). Two genes were significantly upregulated more than 4-fold in the Key west strain compared with Orlando strain, i.e., adam (AAEL004881), and cadherin (AAEL001196). Four genes were significantly downregulated more than 4-fold in the Key west strain compared with Orlando strain, i.e., actin (AAEL001951), adam (AAEL014344), calponin/transgelin (AAEL006922), and tubulin-specific chaperone (AAEL011067).

Nineteen DE cytoskeleton transcripts were dysregulated (9-upregulated and 10-downregulated) between Key West *Ae. aegypti* infected with ZIKV and the Key West control at the 7 dpi (**Table S6C**). These transcripts encoded one activin receptor, one adam, one basic helix-loop-helix zip transcription factor, two cadherins, one calsenilin, one carbonic anhydrase II, one diaphanous protein, one dynactin, one dynein heavy chain, two filmin, one follistatin, one merlin/moesin/ezrin/radixin, one prefoldin, one protein maelstrom homolog, two tubulin alpha chains, and one tubulin-specific chaperone (**Table S6C**).

Nevertheless, all six DE transcripts related to cytoskeleton were significantly dysregulated between Orlando *Ae. aegypti* infected with ZIKV and the Orlando control at the 7 dpi (**Table S6D**). These transcripts encoded one innexin, one muscle lim protein, two myosin, one paramyosin, and three troponins (**Table S6D**).
